# Dissolving microneedles for transdermal delivery of huperzine A for the treatment of Alzheimer's disease

**DOI:** 10.1080/10717544.2020.1797240

**Published:** 2020-07-30

**Authors:** Qinying Yan, Weiwei Wang, Jiaqi Weng, Zhenghan Zhang, Lina Yin, Qingliang Yang, Fangyuan Guo, Xingang Wang, Fan Chen, Gensheng Yang

**Affiliations:** a College of Pharmaceutical Sciences, Zhejiang University of Technology, Hangzhou, China; b Research Institute of Pharmaceutical Particle Technology, Zhejiang University of Technology, Hangzhou, China; c Zhejiang Academy of Medical Sciences, Institute of Materia Medica, Hangzhou, China; d Department of Burns, School of Medicine, Second Affiliated Hospital of Zhejiang University, Hangzhou, China; e Hubei Collaborative Innovation Center for Green Transformation of Bio-Resources, Life Sciences School of Hubei University, Wuhan, China

**Keywords:** Dissolving microneedles, transdermal delivery, huperzine A, Alzheimer's disease

## Abstract

Increasingly attention has been paid to the transdermal drug delivery systems with microneedles owing to their excellent compliance, high efficiency, and controllable drug release, therefore, become promising alternative with tremendous advantages for delivering specific drugs such as huperzine A (Hup A) for treatment of Alzheimer’s disease (AD) yet with low oral bioavailability. The purpose of the present study is to design, prepare, and evaluate a dissolving microneedle patch (DMNP) as a transdermal delivery system for the Hup A, investigating its *in vitro* drug release profiles and *in vivo* pharmacokinetics as well as pharmacodynamics treating of AD. Skin penetration experiments and intradermal dissolution tests showed that the blank DMNP could successfully penetrate the skin with an adequate depth and could be quickly dissolved within 5 min. *In vitro* transdermal release tests exhibited that more than 80% of the Hup A was accumulatively permeated from DMNP through the skin within three days, indicating a sustained release profile. *In vivo* pharmacokinetic analysis demonstrated that the DMNP group resulted in longer *T*_max_ (twofold), longer *t*_1/2_ (fivefold), lower *C*_max_ (3:4), and larger AUC_(0–∞)_ (twofold), compared with the oral group at the same dose of Hup A. Pharmacodynamic research showed a significant improvement in cognitive function in AD rats treated with DMNP-Hup A and Oral-Hup A, as compared to the model group without treatment. Those results demonstrated that this predesigned DMNP is a promising alternative to deliver Hup A transdermally for the treatment of AD.

## 1. Introduction

Alzheimer's disease (AD) is a common degenerative disorders of central nervous system, and is generally considered as the primary cause of dementia in people aged 65 and over (Association, 2017, 2018). AD is characterized by a gradual decline in learning, memory, and executive function, which is the production of amyloid plaques and neurofibrillary tangles in the brain, as well as the loss of neurons associated with memory and learning (Hager et al., 2001; Selkoe & Hardy, 2016; Sevigny et al., 2016). By 2050, the number of AD patients will increase from 47 million to 130 million (Nguyen et al., 2017), which will be a tremendous social and economic burden to the whole world.

Numerous drugs have been designed and developed in the past several decades, including acetylcholinesterase inhibitors (AChEIs) (Galimberti & Scarpini, 2016) and cerebral blood flow and brain metabolism improver (Wu & Wen, 2016). Huperzine A (Hup A), from a Chinese herb, *Huperzia serrata*, is an effective AChEI (Ferreira et al., 2016; Zheng et al., 2016), and has been proved to significantly improve the memory of elderly AD patients (Wang et al., 2006), as well as to enhance the memory and academic performance of young students (Zhang et al., 2002). Currently, commercially available pharmaceutical forms of Hup A include oral dosages such as tablets (Xie et al., 2013; Peng et al., 2019), and capsules (Laicher & Fuchs, 1998) and injections, specifically intramuscular (IM) injection (Zhu et al., 1999). Due to its narrow therapeutic range and rapid metabolism (*t*_1/2 α_=9.8 min, *t*_1/2 β_=247.5 min), Hup A is required to be administered frequently, which not only causes undesirable fluctuation in plasma concentration and probably leads to gastrointestinal side effects such as anorexia, but also brings bad inconvenience in its clinical use (Wang et al., 2007). On the other hand, the IM injection has quite a few side effects, for instance, pain, inconvenience, short curative effect, vascular injury, muscle contracture, nerve damage, and injections site adverse reactions.

As one of the novel drug delivery systems developed recently, transdermal microneedles are capable to deliver drugs by penetrating the skin barrier, the stratum corneum, to improve the overall efficacy of such drugs, to avoid the gastric irritation, to eliminate first-pass metabolism, and to obtain a sustained-release (van der Maaden et al., 2012; Ye, 2018). Currently, five types of microneedles (solid, coating, hollow, hydrogel-forming (Eltayib et al., 2016), and dissolving) have been designed and characterized in depth (van der Maaden et al., 2012), and dissolving microneedle is considered as the safest transdermal delivery system mainly because of the biodegradation and biocompatibility of the utilized needle materials (Pérennès et al., 2006; Park et al., 2016; Choi et al., 2018; Ronnander et al., 2018). Practically, water soluble polymers such as polyvinyl pyrrolidone (PVP) (Li et al., 2020), polyvinyl alcohol (PVA) (Zhang et al., 2020), sodium carboxymethyl cellulose (CMC-Na) (Ono et al., 2017), are commonly applied as appropriate materials for dissolving microneedles. Particularly, sodium hyaluronate (HA) (Kim et al., 2020) is an ideal candidate with the most popularity due to its distinct properties of biocompatibility, biodegradability, non-immunogenicity and frequently used for microneedle manufacturing (Du et al., 2011; Leone et al., 2020; Wang et al., 2020). In a word, the HA dissolving microneedle technology combines the advantages (accurate, convenient, and efficient) of traditional injection and transdermal delivery routes. Meanwhile, disadvantages caused by the syringes can also be avoided, such as the feeling of pain, potential environmental pollution, and the safety risks (Kim et al., 2012).

The present study aims to design and prepare a dissolving microneedle patch (DMNP) to transdermally deliver Hup A, and to evaluate the *in vitro* drug release profiles and *in vivo* pharmacokinetics as well as pharmacodynamics for treating AD.

## 2. Materials and methods

### 2.1. Materials

Polydimethylsiloxane (PDMS, Sylgard 184) was purchased from Dow Corning (Midland, MI). Sodium hyaluronate (750–1000 kDa) was purchased from Lifecore Biomedical (Chaska, MN). Huperzine A powder (Hup A, *M_w_*=242.32, *log p* = 2.70, 99.6% purity, particle size = 120–150 μm) and huperzine B (Hup B, 99.6% purity) were purchased from Zhejiang Wanbang Co., Ltd. (Taizhou, China). Acetylcholine (ACh), superoxide dismutase (SOD), and malondialdehyde (MDA) kits were purchased from Nanjing Jiancheng Biotechnology Institute (Nanjing, China). All other chemicals and solvents were analytical reagent grade.

### 2.2. Animals

Eight weeks old SD rats, 260 ± 15 g, were supplied by the Zhejiang Academy of Medical Sciences (Hangzhou, China). Rats were housed at a temperature range from 20 °C to 25 °C and humidity range from 50% to 60%, and they were fed with commercial aseptic food. All studies were reviewed and approved by the Institutional Animal Care and Use Committee at Zhejiang University of Technology.

### 2.3. Methods

#### 2.3.1. Fabrication and mechanical properties of the DMNP

**Figure 1.**
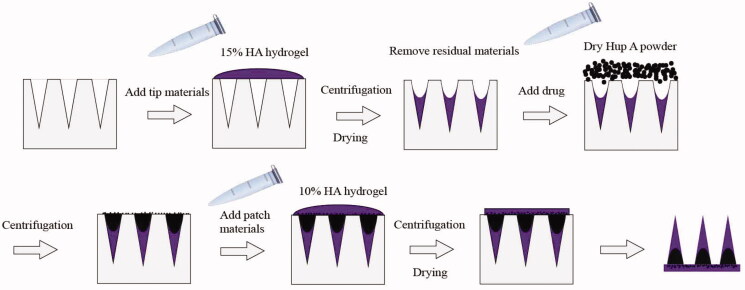
Schematic illustration of Hup A-HA microneedles preparation process.

DMNP was fabricated by the micromolding technology as described with some modifications (Chen et al., 2017; Yan et al., 2017). In this work, HA powder was dissolved by distilled water in Eppendorf tubes with rotation at 25 °C for two days at a Four-Dimensional Rotating Mixer (Kylin-Bell Lab Instruments BE-100, Haimen, China), 15% (w/v) HA gel for the needles preparation and 10% (w/v) HA gel for the patch preparation. The PDMS mold was used to fabricate a patch of conical microneedles (6 × 6) with a height of 500 μm, a tip diameter of 20 μm and a base diameter of 250 μm. The 15% HA gel was added into the mold and centrifuged to fabricate the needles with cavities as reported before (Chen et al., 2017). After dried at room temperatures for overnight, the Hup A powder was loaded into the microneedles base material and cavities by a two-step sandwich (Figure 1), the 10% HA gel was put in the mold to form the patch under centrifugation.

For the qualitative investigation to the hardness of the needles, weights of 50 g, 100 g, 150 g, and 250 g were placed by using a tweezer on top of the formed 4 × 5 DMNPs for 1 min, respectively. After the weights were removed, the deformation of the microneedles was observed by a microscope.

In order to quantitatively test the mechanical properties of the needles, the compression method by texture analyzer (TA.XT Plus, SMS, Godalming, UK) was applied (Dillon et al., 2017). The DMNP with four microneedles (2 × 2) was attached by tape to the movable probe of the analyzer, with the moving speed of 0.1 mm/s, the trigger force of 0.04 N, and the data acquisition rate of 25 pps. The force and displacement were measured and recorded when the uppermost tip of DMNP touched the platform.

#### 2.3.2. Intradermal dissolution, skin penetration of the blank DMNP and skin irritation test of DMNP

To study the skin penetration ability of the DMNP, the blank DMNP was punctured on the abdomen skin of the anesthetized SD rat without hair for 1 min. Then, the rat was sacrificed to dissect the injection site of the skin. After the procedure of fixing and staining by hematoxylin and eosin (H&E), the histological skin slides were scanned and analyzed by a microscopy (Leica DM2500, Wetzlar‎, Germany). The acute irritation test of 5 min treatment by the blank DMNP was also assessed *in vivo*. The state of skin recovery was monitored until the microholes on the skin became nearly invisible to the naked eye. Photographs were received before administration and at 0, 15, 30, 45, 60, 90, 120, 150, and 180 s after administration by a camera. The skin irritation of 24 h DMNP-treated was also recorded by a camera.

In order to investigate the dissolution of the microneedle after penetration into the skin, the blank DMNPs were punctured on the abdomen skin of the anesthetized SD rat without hair, removed after 3 min and 5 min to observe the *in vivo* dissolution of the microneedles by microscope (Olympus SZ-61, Shinjuku City, Japan).

#### 2.3.3. The methods for quantitation of Hup A in DMNP and in serum

Hup A (1 mg) dry powder was added into the cavities of the microneedles as described in section 2.3.1 and enclosed by the 10% HA as the patch (Yan et al., 2017). Hup A in DMNP was analyzed by the method of high-performance liquid chromatography (HPLC). Hup A was dissolved in 5 mL of phosphate buffer, filtered through a 0.45 μm microporous membrane filter, and 20 μL of the filtrate was injected into the HPLC system (Dionex U3000, Lohmar, Germany), with UV detection at 307 nm. The mobile phase consisted of a mixture of acetonitrile and 0.01 M potassium dihydrogen phosphate buffer (10:90, v/v), with a flow rate of 1 mL/min. The column temperature was maintained at 25 °C.

Hup A concentrations in serum were measured by liquid chromatography–mass spectrometry (LC–MS) method (Wang et al., 2004) using LC–MS system (Waters Xevo TQ-S micro, Milford, MA). Chromatographic separation was performed on an ACQUITY UPLC HSS T3 C18 column (1.8 µm particle size, 100 mm × 2.1 mm, Waters, Milford, MA), with acetonitrile–0.1% aqueous formic acid (60:40, v/v) at a flow rate of 2.0 µL/min. Desolvation temperature was set to 350 °C, and 500 L/h nitrogen was used to remove the solvent. The positive ion multiple reaction monitoring (MRM) was used to detect Hup A and Hup B (as an internal standard) with the selected transitions of *m/z* 243.3 → 226.2 and *m/z* 257.3 → 198.2, respectively.

#### 2.3.4. *In vitro* release of Hup A in DMNP

In this study, a modified Franz diffusion cell (Migdadi et al., 2018; Gao et al., 2019; Chen et al., 2020) was used to study the permeability of 1 mg Hup A delivered by microneedle through the abdomen skin of SD rat. The 10% HA mixed with 1 mg Hup A was prepared to be the patch layer as a control group, by the same procedure as mentioned in section 2.3.1. Skin samples obtained from healthy rats with the removal of subcutaneous fat were trimmed to a suitable size with surgical scissors, to fix on a diffusion cell. DMNP-Hup A was punctured into the skin as the test group, while the patch layer loaded with Hup A was applied directly on the skin. Physiological saline was used as a receptor medium, and the temperature was kept at 37 ± 3 °C, with stirring at a constant rate of 500 rpm. All receiving chamber fluids were withdrawn at appropriate intervals and then supplemented with an equivalent amount of physiological saline. The samples were assayed by HPLC after filtered through a 0.45 μm filter.

#### 2.3.5. Pharmacokinetic analysis

Eighteen SD rats were allowed to acclimatize for seven days prior to experimentation. The flank hair of the rats was removed after anesthetized. They were divided into three groups of six rats each: (a) one group received oral administration of Hup A and (b) two groups received DMNP administration of Hup A with a different dosage. For the oral group, 0.5 mg Hup A solution was inserted into the stomach using oral gavage (ig). For the DMNP groups, DMNP-Hup A with a drug loading of 0.5 mg (a half patch, 3 × 6) and 1 mg (a single patch, 6 × 6) were then respectively applied and stuck to the naked flank skin with adhesive tapes (3M Transpore tapes, 3M Healthcare, Loughborough, UK). Blood samples were taken from the orbital sinus at pre-defined time intervals: 0, 1, 2, 3, 4, 6, 8, 12, and 24 h with 1 mL collected at each sampling point. To separate plasma from the blood, the tubes were centrifuged at 3000 relative centrifugal force (RCF) for 5 min at 4 °C. 0.5 mL of plasma was subsequently aliquoted into 1.5 mL Eppendorf tubes and stored at −40 °C until used.

Prior to analysis, frozen samples were thawed at 37 °C in a water bath and vortexed briefly. For the analysis, 20 μL internal standard of Hup A methanol solution (1 μg/mL), 100 μL sodium hydroxide solution (1 mol/L), and 5 mL re-distilled dichloromethane solution were added into the samples. After centrifugation at 5500 rpm for 10 min and vortex mixing for 5 min, the organic phase was transferred to another glass tube and evaporated to dryness at 40 °C under a gentle stream of nitrogen. The residue was reconstituted in 100 μL methanol, and centrifugation at 10,000 rpm for 5 min, then the supernatant was collected and injected into the LC–MS system. The separation was performed using a Waters HSS T3 column (100 mm × 2.1 mm, 1.8 μm) in an Acquity^TM^ ultra performance liquid chromatography (UPLC) system. Acetonitrile and water containing 0.1% of formic acid (60:40 v/v) were used as mobile phase A and mobile phase B, respectively. The flow rate was 2.0 μL/min. The temperature for the column was 30 °C. The analyte was quantified using a Waters XEVO TQ-S micro triple quadrupole mass spectrometer equipped with an electro-spray ionization (ESI) source. Multiple reactions monitoring (MRM) scan type was used in the positive scan mode to increase the specificity of the analysis.

#### 2.3.6. Pharmacodynamic evaluation

In this work, the Morris water maze test was used to investigate the effects of Hup A-DMNP on the acquisition and memory impairments (Vorhees & Williams, 2006). The qualified SD rats were randomly divided into four groups (six rats per group), and given the following treatment daily for 11 days: (1) the microneedle group was given DMNP-Hup A (half patch, 3 × 6, containing 0.5 mg Hup A) per rat, once a day (2) the oral group was given 1 mL Hup A (0.5 mg/mL) orally per rat, once a day, (3) the model group and (4) the control group were given 1 mL physiological saline orally (ig), once a day, respectively.

Since the 6th day, the rats were trained under the conditions of the platform to familiarize themselves with the pool environment. Then the positioning navigation test was carried out four times a day lasting for six days. Briefly, the rats were placed in the water facing the pool wall at one of four selected starting points (northwest, northeast, southwest, and southeast pole), and had to learn a new location of the submerged platform within 120 s. On the 11th day, the injection of scopolamine hydrobromide (1 mg/kg, ip) was performed 30 min after the daily treatment, except for the control group, to create a short-time memory defect AD model (Wang et al., 2007). Forty-five minutes later, a four-quadrant water maze test was performed to record the escape latency (Dhingra & Soni, 2018).

The experimental rats were sacrificed immediately after the end of the water maze test. The brains were quickly removed and the cerebral cortex was separated on the icebox. After washed by saline solution, the 10% brain homogenate was prepared using 0.9% sodium chloride solution, then centrifuged for 10 min at 3500 rpm (0 °C). Finally, the supernatant was collected for the subsequent kit verification of ACh, SOD, and MDA. The absorbance was recorded at 550, 450, and 532 nm, respectively.

#### 2.3.7. Statistical analysis

Data were analyzed by one-way ANOVA among multiple groups or a Student's *t*-test between two groups with Prism Graph Pad software (Graph-Pad Software, La Jolla, CA). A value of *p*< 0.05 was considered statistically significant.

Pharmacokinetic data analysis was performed using a non-compartmental method, i.e. statistical moment analysis, and all pharmacokinetic parameters were calculated using the Drug and Statistic (DAS) 2.0 pharmacokinetic software (Chinese Pharmacological Association, Beijing, China). Parameters of AUC_(0–∞)_ and plasma half-life (*t*_1/2_) were calculated and reported. The maximum measured plasma concentrations (*C*_max_) and the corresponding times (*T*_max_) were obtained directly from the raw data after the last dose was given.

## 3. Results and discussion

### 3.1. The morphology of the blank DMNP

**Figure 2.**
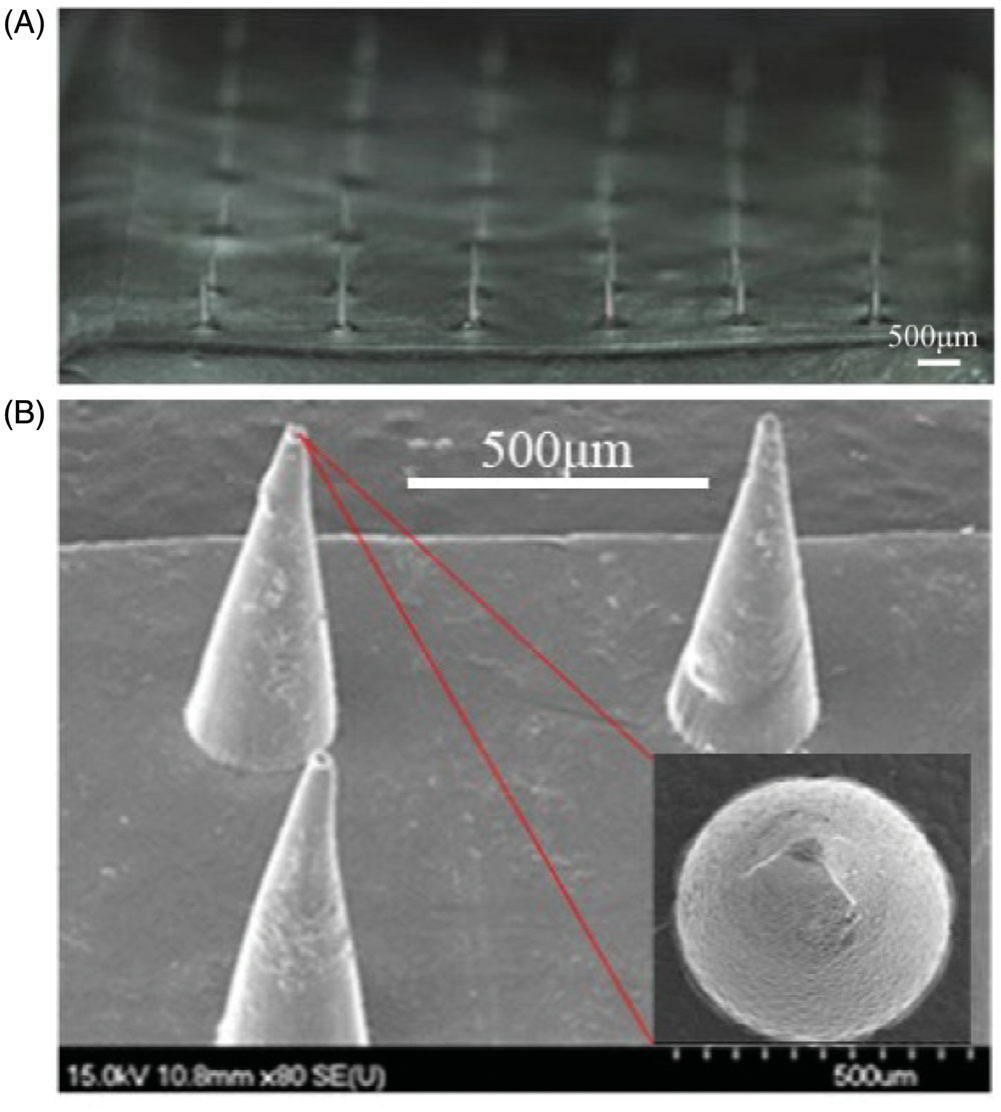
The morphology of the blank DMNP. (A) The overall appearance of the 6 × 6 DMNP. (B) SEM image of the HA DMNP, with a height of 489 ± 7.64 μm and a base diameter of 242 ± 6.03 μm. Scale bar, 500 μm.

The blank DMNP was observed under the SEM. As shown in Figure 2(A), it was a square patch with 36 needles, of which height was 489 ± 7.64 μm and base diameter was 242 ± 6.03 μm. The shape of the needle was conical, with a smooth surface and a sharp tip (Figure 2(B)).

### 3.2. The mechanical properties of the blank DMNP

**Figure 3.**
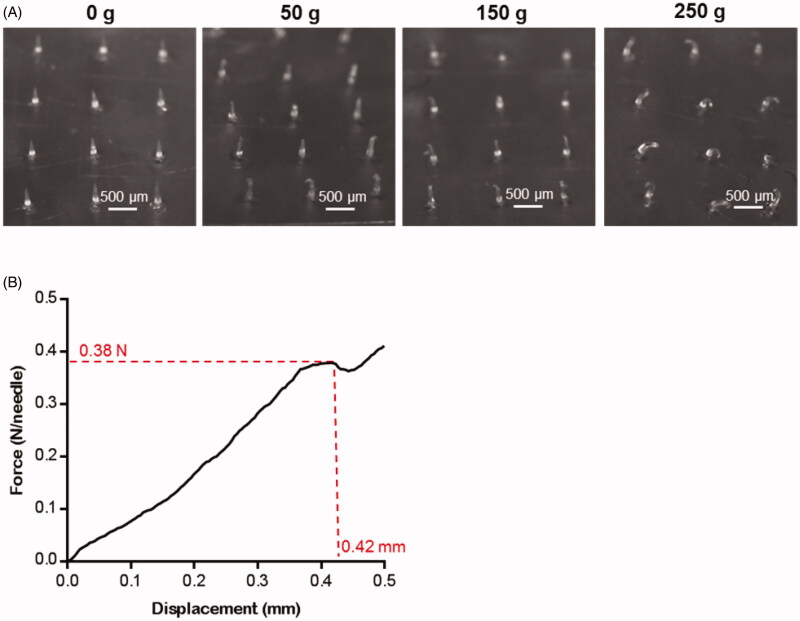
The mechanical properties of the blank DMNP. (A) Morphological changes of DMNP (4 × 5) loaded with different mass weights (0, 50, 150, and 250 g). Scale bar, 500 μm. (B) Representative pressure-displacement curve of the DMNP (2 × 2) measured by texture analyzer.

For the qualitative investigation to the hardness of the needles, the weight-bearing experiment was carried on. 50 g, 150 g, and 250 g tweezers were placed on the 4 × 5 DMNPs respectively for 1 min. As shown in Figure 3(A), the shape of microneedles scarcely changed under the weight of 50 g, and still maintained the upright state when the weight was 150 g. While under the weight of 250 g, which force can be calculated to 0.12 N/Needle, all microneedles were nearly half bending. However, no broken fragments were observed, showing excellent hardness and flexibility of the DMNP.

The compression method by texture analyzer was applied in order to quantitatively test the mechanical properties of the needles. From the experimental results as shown in Figure 3(B), the force was almost linear up to 0.38 N/Needle with displacement at 420 μm till the microneedle was broken, while the displacement was 170 μm at the force of 0.12 N/Needle, which was corresponded with the qualitative investigation result as mentioned above. According to the previous study, a force of 150 mN/Needle was needed to penetrate human skin for microneedle with a tip diameter of 24 μm (Yu et al., 2017). Thus, the DMNP in this study should have enough mechanical strength for transdermal delivery of Hup A.

### 3.3. Skin penetration, intradermal dissolution of the blank DMNP, and skin irritation test of blank DMNP

**Figure 4.**
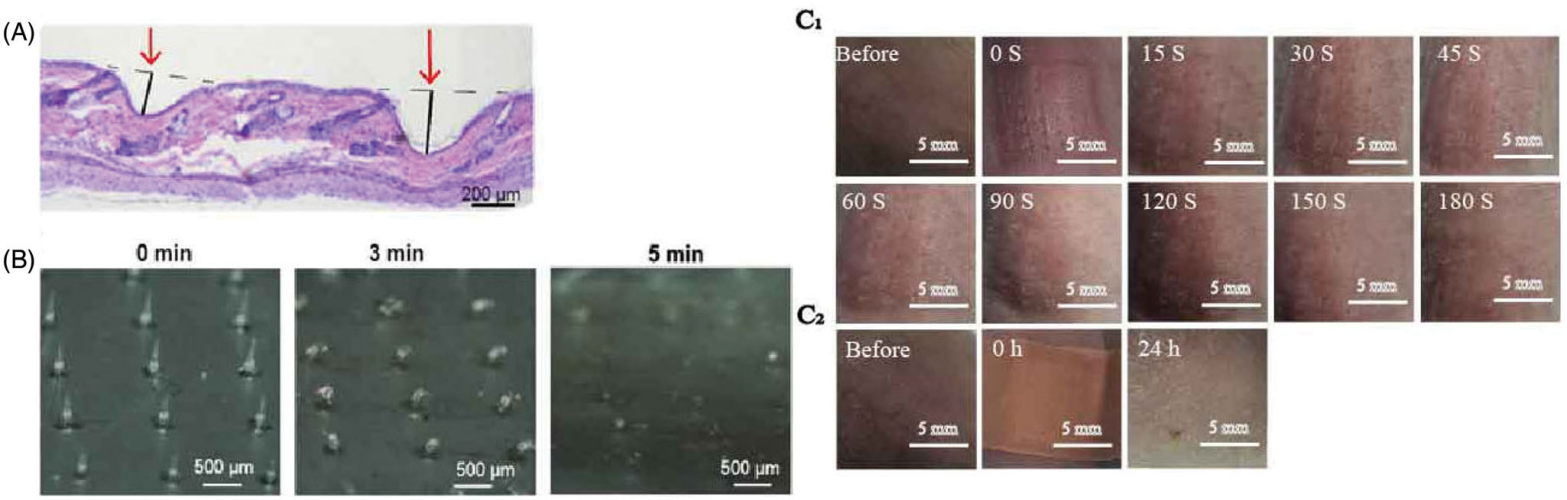
Skin penetration and intradermal dissolution of the blank DMNP. (A) Histological skin sections were prepared after insertion of DMNP to show skin penetration, scale bar, 200 μm. (B) The morphology of DMNP taken out from the rat skin at different time intervals after penetration, scale bar, 500 μm. (C) Images of DMNP-treated rats dorsal skin (C_1_, at different time intervals; C_2_, after 24 h DMNP-treated), scale, 5 mm.

The DMNP could be pierced through the rat epidermis as confirmed histologically, with the depth of more than 200 μm (Figure 4(A)), which demonstrated the drugs loaded in the DMNP could be delivered into skin effectively. As shown in Figure 4(B), the tip of the DMNP was half dissolved in 3 min and disappeared after 5 min, which represented the DMNP here could be quickly dissolved and successfully release its cargo in 5 min. As shown in Figure 4(C_1_), the puncture marks gradually disappeared over time and the spots on skin finally became invisible 180 s later. After 24 h DMNP-treated, no obvious erythema and swelling were occurred. Those results indicated that the prolonged adherence did not cause severe inflammation for the reason that the microneedle materials (HA) possess a satisfactory biocompatibility, yet did cause a little redness resulted from the used bandage, which would be replaced with a more suitable material in our further studies.

### 3.4. *In vitro* transdermal release profile of Hup A

**Figure 5.**
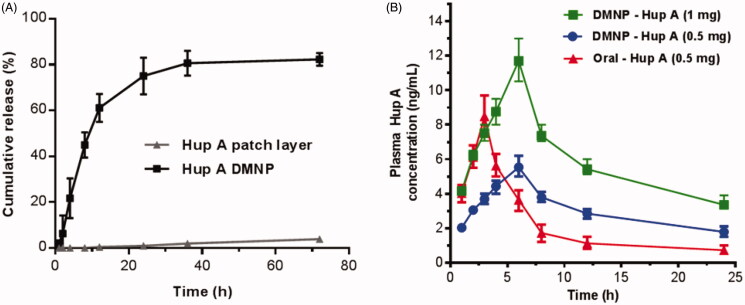
*In vitro* and *in vivo* transdermal release profile of Hup A. (A) *In vitro* cumulative permeation curve of Hup A (1 mg) loaded in patch layer and DMNP, which were measured by a modified Franz diffusion cell. (B) *In vivo* release curve of Hup A by different routes and dosages in rats. Data are expressed as the mean ± SD (*n* = 6).

The Hup A was loaded in both the tip portion and the backing layer of the DMNP, which dramatically increased the drug loading and prolonged the release of the drug. As shown in Figure 5(A), notably, Hup A in DMNP performed an initial sharp burst release in the first 12 h, followed by a moderate release in the following 24 h, and stabilized gradually in the last 36 h. A set of interacting phenomena could explain this result as previously reported (Chen et al., 2013). When DMNP were inserted into the skin, Hup A located in the tip portion of DMNP may be rapidly dissolved by skin interstitial fluid, resulting in the initial burst release and the HA-induced hydration of the epidermis, which could contribute to drug retention in the skin and slow down the permeation process (Brown & Jones, 2005). Subsequently, Hup A located in the backing layer of DMNP may gradually diffuse from the HA matrix, as well as the poor solubility of the Hup A in skin, providing sustained release for nearly three days. During this period, approximately 80% of the Hup A was cumulatively released, in contrast, Hup A in the control group (loaded in patch layer) showed a poor release profile with nearly 5% cumulative release in three days. The *in vitro* release result clearly indicated that HA could be a suitable material for sustained release of Hup A, and DMNP could significantly improve the permeation rate of the transdermal delivery of Hup A.

### 3.5. Pharmacokinetic analysis

The concentration of Hup A in the plasma samples was detected with a validated HPLC–MS method (Wang et al., 2004). The main pharmacokinetic parameters of Hup A after being given ig and td are shown in Table 1. Compared with oral administration of the same dose of Hup A (0.5 mg), the DMNP group resulted in longer *T*_max_ (twofold), longer *t*_1/2_ (fivefold), lower *C*_max_ (3:4), and larger AUC_(0–∞)_ (twofold). When considering the dosage factor, there were no significant discrepancies in the data of *T*_max_ and *t*_1/2_ between the low and high dose groups (*p*> 0.05), while the values of *C*_max_ and AUC_(0–∞)_ from different groups were dose-related. As shown in Figure 5(B), the mean Hup A serum concentrations increased during the first 6 h after the administration of DMNPs, then reached the mean maximum concentrations, and decreased steadily in the next 18 h. Due to the continued absorption of Hup A in skin from the DMNP groups, a slower rate of disappearance was performed than the oral groups. Compared to previous studies, which focused on the oral administration (Peng et al., 2019) and injection (Chu et al., 2006), respectively, the HA DMNP proposed by the present study is more advantageous which not only produced a higher bioavailability but also accomplished a sustained release with the strategy of the DMNP.

**Table 1.** Pharmacokinetic parameters after different administration routes of Hup A in rats.

Parameters	Oral (0.5 mg)	DMNP (0.5 mg)	DMNP (1 mg)
*C*_max_ (ng/mL)	8.48 ± 0.91	5.53 ± 0.53	11.70 ± 0.96
*T*_max_ (h)	3.00 ± 0	6.00 ± 0	6.00 ± 0
*t*_1/2_ (h)	3.44 ± 0.40	15.21 ± 2.09	14.32 ± 0.75
AUC_(0–∞)_ (ng·h/mL)	53.32 ± 8.73	110.88 ± 17.95	209.59 ± 18.83

Mean ± SD, *n* = 6.

One common disadvantage of the traditional transdermal system is the low bioavailability. Therefore, sufficient medicine is required as the penetration force, and a large amount of the medicine still remains after wearing (Ye et al., 2008; Wu et al., 2011; Amjadi et al., 2018; Lee & Prausnitz, 2018). Results in this study indicated that DMNP can prolong the elimination of *t*_1/2_ and enlarge the area under the curve (AUC), presenting a higher relative bioavailability value than that of oral administration. Combining with our previous research findings (Chen et al., 2017), it can be concluded that dissolving microneedle technology has the advantages of delivering drugs into the skin interstitial fluid with a controlled and continuous release, reducing the frequency of administration, and improving the compliance of patients, which are very important to these drugs of short half-life.

### 3.6. Pharmacodynamic evaluation

**Figure 6.**
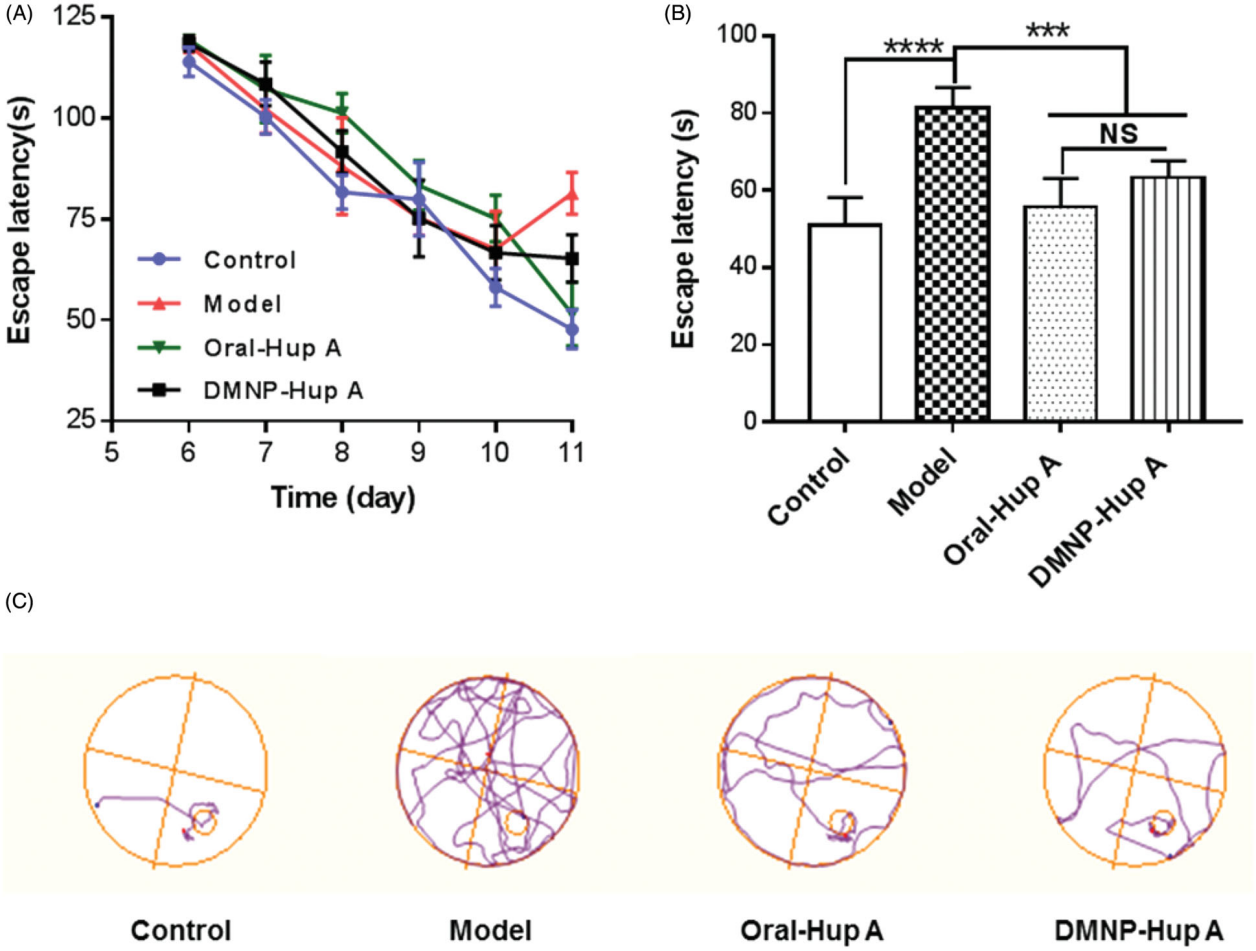
Investigation of animal behavior in different groups. (A) Escape latency in the Morris water maze test on different training days. (B) Escape latency after modeling in different experimental groups. (C) Representative swim paths. Data are expressed as the mean ± SD (*n* = 6). ***p< 0.001, ****p< 0.0005, and NS, no significance.

The learning and memory performances in different groups were assessed by the Morris water maze task. As shown in Figure 6(A), positioning navigation test (day 6 to day 10) showed that as the number of training days increased, the escape latency of each group gradually decreased, indicating that each group of rats has gradually learned to find a platform in learning and training. There was no significant discrepancy in learning and memory between any of the groups. On the 11th day, rats were injected intraperitoneally with scopolamine hydrobromide to establish a short-time memory deficit model. Forty-five minutes later, the positioning navigation test (11th day) was performed. Figure 6(B) illuminates that a significant discrepancy of escape latency between the following groups: Control vs. Model (*p* < 0.0001), Oral-Hup A and DMNP-Hup A vs. Model (*p* < 0.001), indicating that the rat's multiple memory-related behaviors were affected after intraperitoneal injection of scopolamine hydrobromide, and the treatment of Hup A in oral as well as in DMNP way could partially reverse the memory deficit with no significant discrepancy (*p* > 0.05). Likewise, according to Figure 6(C), the typical swimming tracks indicated that the rats in Model groups often searched the platform in an inappropriate manner, resulting in a longer latency for the positioning platform than the other groups, and the DMNP-Hup A group had the similar effect as the Oral-Hup A group.

**Figure 7.**
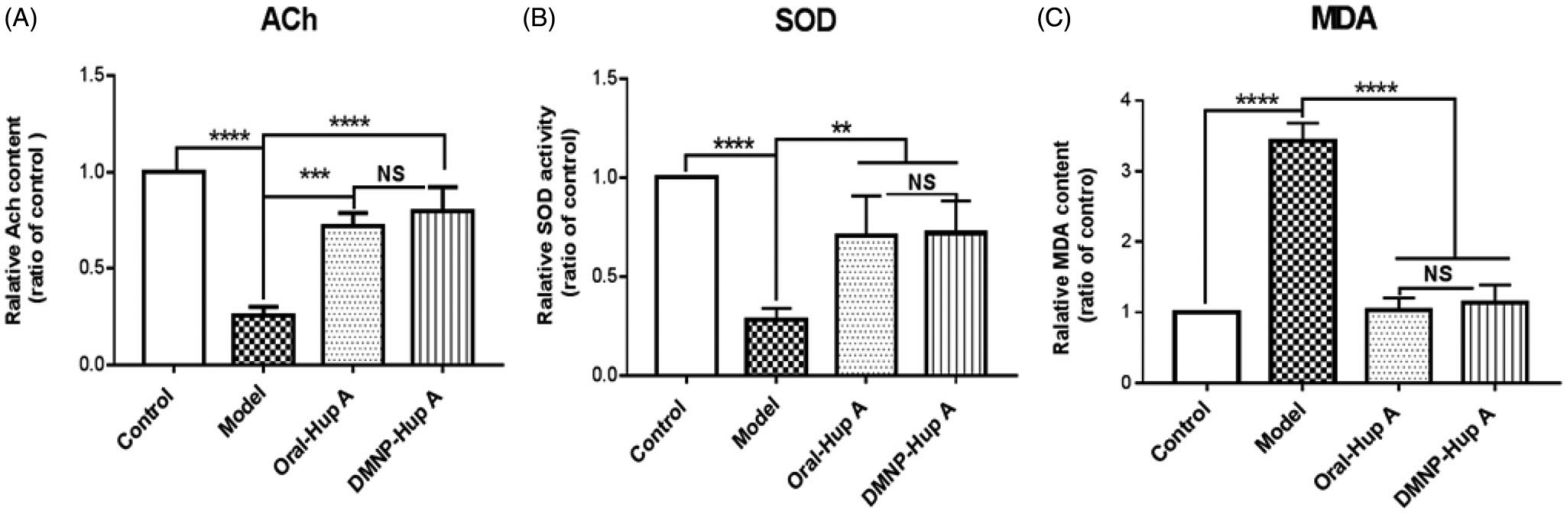
Effect of DMNP-Hup A on (A) ACh content, (B) SOD activity, and (C) MDA level in rats. Data are expressed as the mean ± SD (*n* = 6). **p< .05, ***p< .001, ****p< .0005, and NS, no significance.

As is well known, ACh is a cholinergic neurotransmitter that participates in information transmission and processing. Numerous studies have confirmed that enhancing ACh content can improve cognitive impairment in rats with dementia (Foyet et al., 2015; Perez-Lloret & Barrantes, 2016). In addition, oxidative damage of biomacromolecules such as lipoprotein in the cellular membranes is always related to oxidative stress. Elevated MDA is considered to be a specific indicator of lipid peroxidation during oxidative damage (Tsikas, 2017). Moreover, oxidative damage can also destroy the antioxidant defense system, accompanied by decreasing SOD activities. The levels of ACh, SOD, and MDA in different groups from our current work are displayed in Figure 7. As shown in Figure 7(A,B), there was a marked decreasing of ACh content and SOD activity in the Model group (*p*< 0.01), as compared to the Control, Oral-Hup A, and DMNP-Hup A groups. In contrast, the level of MDA content in the Model group was increased obviously (*p*< 0.001) with threefold higher than the other groups (Figure 7(C)). These data above illustrated that in the Model group, declining ACh content and oxidative damage (increased MDA content, decreased SOD activities) lead to the cognitive disturbance consistent with Figure 6(C). Finally, the phenomenon was significantly reversed after the treatment of Hup A both in Oral and DMNP ways, with no significance (*p*> 0.05). Collectively, these findings suggest that dissolving microneedle delivery of Hup A could effectively improve the cognitive impairment in rats with dementia.

Moreover, as the *in vitro* release profile (Figure 5(A)) showed, about accumulatively 80% of Hup A released in 24 h and then enter a stable phase. It can be speculated that 20% of the 0.5 mg Hup A still remain in the patch, owing to the gaps between microneedles which may prevent this part of the drug from being delivered to the skin. However, DMNP-Hup A can also achieve a satisfactory therapeutic effect with a smaller drug dose and fewer administration times than the oral way.

## 4. Conclusions

Presently, the marketed pharmaceutical forms of Hup A are tablets, capsules, and im injection. However, frequent administration and potential side effects, such as anorexia, are the main problems in oral routes. On the other hand, the im injection also has quite a few side effects, such as pain, inconvenience, vascular damage, nerve injury, injections site reactions, etc. Transdermal drug delivery strategy with dissolving microneedle is not only capable to significantly improve the bioavailability of the Hup A comparing to its oral administration, but also able to accomplish a desirable sustained release in a painless and minimally invasive manner. This study designed and developed a new strategy to transdermally deliver Hup A by DMNP, with advantages of high-dose Hup A loading, sustained-release, efficient transdermal delivery, and satisfactory therapeutic effect. The data indicated that DMNP-Hup A could clinically penetrate the skin in a minimal invasive, to deliver drugs into the skin interstitial fluid with a controlled and continuous release, and the effect of this method was similar or even better than that achieved using the oral way.
